# Effect of psoralen on the expression of PPARγ, osteocalcin, and trabecular bone area in rabbits with steroid-induced avascular necrosis of the femoral head

**DOI:** 10.1186/s13018-018-1054-0

**Published:** 2019-01-08

**Authors:** Huiying Li, Dongfang Meng, Xiaorui Zhang, Dong Yuan

**Affiliations:** Orthopedics Department, The First Affiliated Hospital of Henan University of Chinese Medicine, 19 Renmin Road, Zhengzhou, 450000 Henan China

**Keywords:** Steroid-induced avascular necrosis of the femoral head, Psoralen, Trabecular bone area, Bone marrow, Osteocalcin, PPARγ

## Abstract

**Objective:**

Psoralen is a natural plant toxin which has the function of protecting fungi, insects, and herbivores. In this study, we aim to investigate the effect and mechanism of psoralen on steroid-induced avascular necrosis of femoral head (SANFH).

**Methods:**

Thirty rabbits were randomly divided into blank group (*n* = 10), model group (*n* = 10), and experimental group (*n* = 10). Rabbits in blank and model groups were treated with normal saline, and rabbits in experimental group were treated with psoralen. Total RNA of bone marrow was extracted by trizol, and the mRNA expression of PPARγ and osteocalcin were detected by q-PCR. Then, the mRNA expression of PPARγ and osteocalcin in the three groups were compared. Western blot was used to detect the PPARγ protein expression in the bone of rabbits. ELISA was used to measure the osteocalcin protein.

**Results:**

The mRNA expression of PPARγ in model group significantly increased compared with blank group. The mRNA expression of osteocalcin in model group decreased compared with blank group. However, the mRNA and protein expressions of PPARγ in experimental group were significantly decreased compared with the model group. The protein expressions of osteocalcin increased compared with the model group. There was no significant difference of trabecular bone area (TBA) between experimental and blank groups (*P* > 0.05). TBA in model group was lower than the experimental group (*P* < 0.05). There was no significant difference of TBA between experimental and blank groups (*P* > 0.05).

**Conclusion:**

This research confirms that psoralen plays a positive role in the rehabilitation of SANFH.

## Background

Avascular necrosis of the femoral head (ANFH) can occur in patients who receive hormonal therapy, causing steroid-induced avascular necrosis of the femoral head (SANFH) [1]. SANFH is considered to be one of the most common hip pain diseases. The disease can be divided into two categories: traumatic and non-traumatic. The former is mainly caused by hip trauma, such as femoral neck fracture and hip dislocation. The latter is mainly caused by the use of corticosteroids and alcoholism in China [2]. The causes of high-dose hormones affecting blood viscosity are congenital dysplasia of hip vessels, sclerotin, and biologic anatomy. Increased blood viscosity may cause vasospasm of the femoral head, even lead to insufficient blood oxygen supply of the femoral head, and then lead to loss of osteocyte activity and apoptosis [3]. The situation does not have significant improvement in the short term, resulting in fracture and even microfracture of the trabecular bone in the weight-bearing area of the femoral head.

ANFH that caused by the use of hormones is non-traumatic, and its pathogenesis is difficult to detect. The main symptom is pain of hip and groin, which is easy to be neglected and misdiagnosed; thus, it is easy to miss the best time of treatment. At present, all kinds of diseases are treated by hormone pulse therapy to cure patients and control their condition. However, this treatment can lead to long-term accumulation of hormones and significant increase in blood viscosity and vascular infarction in patients leading to the bone formation decreased, the bone trabecular turned sparse, the sclerotin dropped, the fracture fragments accumulated rarefaction of bone; and finally, steroid-induced femoral head formed [4]. ANFH is difficult to find in its early stage, and due to the relatively poor medical conditions in some areas, some patients may be misdiagnosed and delayed to be cured. In addition, the disease course is long, and its late disability rate is high. Therefore, in order to further clarify the etiology, it is necessary to do early diagnosis and take the treatment as soon as possible, so as to alleviate the suffering of patients.

Psoralen is the dry and ripe fruit of *Psoralea corylifolia* L. Pharmacological studies have found that psoralen has anti-cancer activity which can enhance immunity and promote bone growth, and it also plays the role of bacteriostasis and anti-psoriasis [5]. Some research reported that psoralen promotes cartilaginous extracellular matrix (ECM) synthesis, as well as increased cartilaginous gene expression, and it may be a useful bioactive component for activating the cartilaginous cellular functions of chondrocytes [6]. Based on the present condition, an in-depth study on SNAFH was to be made, with the advantages of traditional Chinese medicine combined with the advanced medical research methods. On the basis of the research, safe and effective drugs can be made to apply in clinic, reduce the patients’ economic burden and pain, at the same time reduce disability rate, and promote the development of traditional Chinese medicine as well as modern scientific theory.

## Materials and methods

### Experimental animal

Approval for the experiment was obtained from the First Affiliated Hospital of Henan University of Chinese Medicine, (Approval number: YFYDW2016032), and all procedures were conducted in conformity with the guidelines and regulations of the First Affiliated Hospital of Henan University of Chinese Medicine. A total of 30 healthy adult New Zealand big ear white rabbits were selected in this research, male and female each 15, weighing 2.5–3.0 kg for each rabbit. The rabbits were purchased from Ji’nan Jinfeng Experimental Animal Center (license No. SCXK (Lu) 20140006) and were fed by pellet feed, each rabbit a cage. Feed was purchased from Henan Animal Experiment Center (license No. SCXK (Yu) 2015-0005). Psoralen was purchased from Anhui Pu Ren Chinese Herbal Medicine Co., Ltd. (batch No.: 160627).

### Methods

#### Grouping and intervention

The New Zealand white rabbits were fed in different cages and were freely to drink water with the normal diet. The humidity was about 50%. The room temperature was controlled between 23 °C and 25 °C. The ventilation was good, and drugs were administrated to the rabbits if there is no obvious abnormality after 1 week’s feeding.

**Fig. 1:**
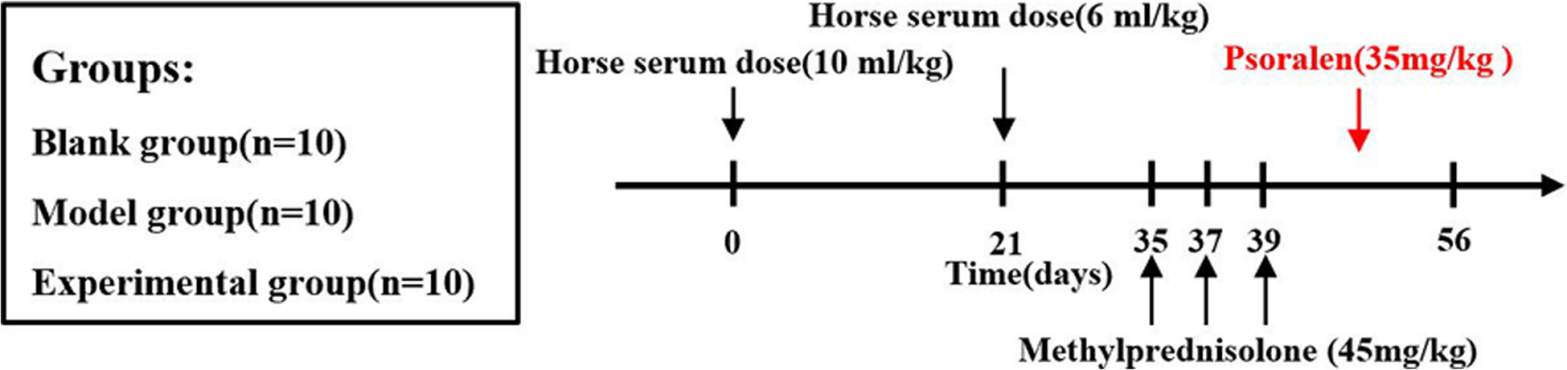
Grouping

The selected rabbits were randomly divided into three groups after being quarantined and observed for 1 week and were determined if there was no abnormality: blank group (*n* = 10), model group (*n* = 10), and experimental group (*n* = 10). After conducting the *t* test to rabbits’ weight and number of male and female in three groups, as well as confirming there was no significant difference, the experiment was started (Fig. 1). All rabbits in the model group were prevented from anaphylactic shock and routinely given horse serum (Zhengzhou Yi Kang Bioengineering Co. Ltd) to establish osteonecrosis of the femoral head model. The rabbit model of alcohol-induced ONFH was successfully prepared by intravenous injection of horse serum. Compared with the pure alcohol gastric perfusion, the success rate of this method was higher, and the pathological changes of ANFH were more obvious. The lesions were concentrated in the subchondral area of femoral head [7]. The specific applications were as follows: the first application of the horse serum dose was 10 mL/kg, through the ear vein injection into the blood, fed 3 weeks conventionally after the drug administration; the second time was also administered by the ear vein, and the horse serum was 6 mL/kg. Then, the rabbits were still fed regularly in the last 2 weeks, and the rabbits should be observed to see whether they have any changes. Two weeks later, we conducted hormone modeling with methylprednisolone (Hubei Fangleda Biochemical Co. Ltd) intraperitoneal injection, and sustained given medication for 3 days according to the dose of 45 mg/kg, injected once a day. During the period of hormone administration, 100,000 units of penicillin were administered to each rabbit every day for seven consecutive days to prevent infection. The rabbits of blank group were fed as usual. In the experimental group and blank group, every rabbit was given (6 g L^−1^) 35 mg/kg psoralen (Anhui Pu Ren Chinese Herbal Medicine Co. Ltd) extract daily. Rabbits in each group were given intragastric administration for 8 weeks. They were weighed once a week and all of them were fed normally.

#### Materials

Eight weeks later, rabbits in each group completed treatment with drugs. Their skins were incised after intravenous anesthesia through the ear vein, and the bilateral femoral heads were exposed after the tissue separation. Visual observation of the femoral head shape was conducted to find out whether the bones were loose or not and whether the weight-bearing area of the femoral head was collapsed or not. Bilateral femoral heads were cut, and the femoral heads were incised from the middle of coronal plane. We rapidly aspirated the red bone marrow with a 5 ml syringe, and it was placed in a sterile centrifuge tube filled with DMEM culture medium.

Extraction of osteocalcin mRNA and PPARγ mRNA was done as follows: the total RNA of bone marrow was extracted by trizol method, and the expression of PPARγ mRNA and osteocalcin mRNA was detected by fluorescence quantitative PCR instrument according to the reagent box instruction. The expression of PPARγ and osteocalcin protein was detected by western blot and ELISA.

The ratio of trabecular bone area (TBA) was obtained as follows: all HE-stained slices were observed × 40 in an optical microscope, in randomly selected five fields of vision, and photographed. Then, the Motic Image Advanced 3.2 analysis software was used to calculate the TBA in the field of vision, calculate the fraction of TBA, and take the average of the TBA ratio.

#### Statistical analysis

All the data were analyzed and processed by SPSS19.0 statistical analysis software. The measurement data were expressed by ($\overline{\mathrm{x}}$ ± s), and one-way ANOVA was used to analyze comparison between groups. The LSD method was used to compare the variances between groups. Once the variance was not uniform, we then immediately replaced LSD method with Dennett’s T3 method. There was a significant difference at *P* < 0.05.

## Results

### Psoralen improved the pathological changes of steroid-induced osteonecrosis of the femoral head

**Fig. 2:**
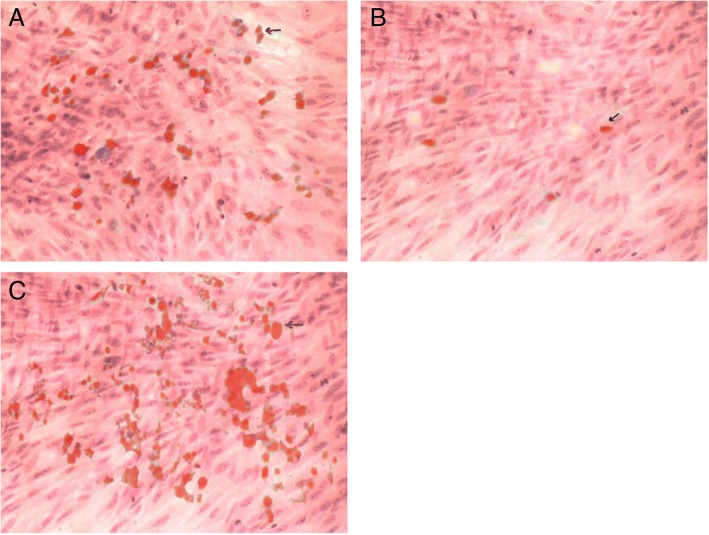
Adipocytes. **a** Oil red O staining × 100 of experimental group. **b** Oil red O staining × 100 of blank group. **c** Oil red O staining × 100 of model group

The morphological changes of adipocytes were observed as follows: when primary cells were inoculated for about 24 h, the cells began to adhere to the wall, showing a circular undifferentiated state. After 3 to 4 days, the cells became spindle deformed and the volume gradually increased, forming fibrous cell morphology and began to split and proliferate. After 7 days, the volume of cells increased, which was fusiform, triangular, or irregular. After 10 days, the proliferation of cells was significantly accelerated, with most of them clustered along a certain direction and with a few irregular arrangements. After 14 to 16 days, the cells aggregated into layers and showed colony growth (Fig. 2a blank group, b model group, c experimental group).

The average diameter of adipocytes in the experimental group and blank group was at a low level (38.00 ± 2.94 and 29.50 ± 3.10), without significant difference between the two groups (*P* > 0.05). The average diameter of adipocytes in the model group was higher (52.25 ± 4.78), with a significant difference compared with the experimental group and the blank group (*P* < 0.05).

**Fig. 3:**
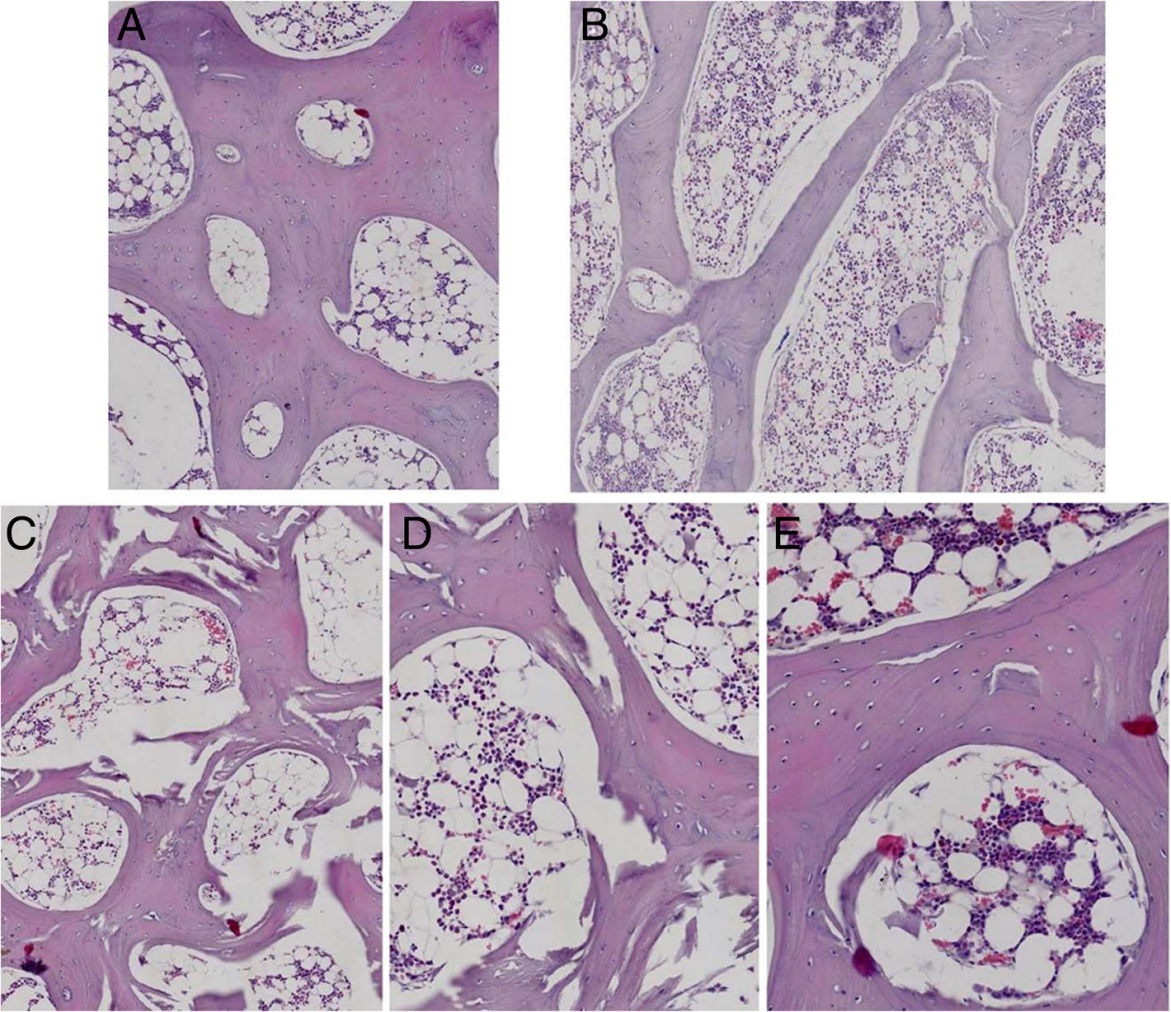
Comparison of three groups of trabecular bone after treatment with psoralen: **a** trabecular bone of blank group was arranged neatly and the bone pits were rare (HE*100); **b** adipose cells in the bone marrow cavity of the blank group were rare (HE*100); **c** trabecular bone were getting thinner and some of them were fractured in the model group (HE*100); **d** the bone cells in model group were deformed and sparse, and the fat cells in the marrow cavity increased significantly (HE*200); **e** bone trabecular of experimental group were arranged neatly (HE*200)

The pathological change of HE staining is shown in Fig. 3 (a blank group, b model group, c experimental group). Psoralen significantly improved the structure of avascular necrosis of the femoral head in experimental group.

### Psoralen decreased PPARγ and increased osteocalcin expressions in steroid-induced osteonecrosis of the femoral head

The relative expression of PPARγ mRNA and osteocalcin mRNA was analyzed by comparison of the 2^− △△Ct^ [5]. GAPDH was used as an internal gene to calculate the △Ct values of each group (the Ct_target gene_ subtracted the Ct_internal gene_), and the △△ Ct value was obtained by comparison between groups (the two groups of △Ct values subtracted). The changes of comparative objective genes between groups were analyzed by the value of 2^− △△Ct^: 2^− △△Ct^ ≥ 2, the expression of comparative target gene between the groups was significantly increased; and 2^− △△Ct^ ≤ 0.5, the expression of comparative target gene between the groups was significantly decreased. The results showed that compared with the blank group, the relative expression of PPARγ mRNA in the model group was significantly increased, and the expression of osteocalcin mRNA was significantly decreased. Compared with the model group, the PPARγ mRNA of the experimental group was significantly decreased and the expression of osteocalcin mRNA was significantly increased (Table 1).

**Table 1:** Expression of PPARγ mRNA and osteocalcin mRNA in each group ($\overline{\mathrm{x}}$ ± s)

Group	GAPD (Ct value)	PPARγ			Osteocalcin		
		Ct	△Ct	2^− △△Ct^	Ct	△Ct	2^− △△Ct^
Blank group	24.00 ± 2.32	33.54 ± 0.99	9.54 ± 1.50		28.37 ± 1.96	4.37 ± 2.03	
Model group	27.06 ± 2.13	34.28 ± 0.46	7.21 ± 1.77	5.02	32.51 ± 1.97	5.44 ± 1.07	0.47
Experimental group	25.47 ± 2.65	33.95 ± 1.69	8.48 ± 1.55	0.42	29.70 ± 2.37	4.23 ± 1.37	2.32

Note: There are significant differences in the same column data for different characters

From Table 2, TBA, the single factor variance test showed that there was a significant difference between the groups in TBA (*P* < 0.05). Compared with blank group and experimental group, the TBA of model group was at the lowest level with a significant difference (*P* < 0.05). TBA of experimental and blank groups were high with no significant difference between groups (*P* > 0.05).

**Table 2:** Effect of psoralen on trabecular bone area in each group ($\overline{\mathrm{x}}$ ± s) (*n* = 10)

Group	Number	Ratio of trabecular bone area
Blank group	10	45.23 ± 3.53
Model group	10	34.72 ± 2.98
Experimental group	10	41.45 ± 3.18

Note: There are significant differences in the same column data for different characters (*P* < 0.05)

**Fig. 4:**
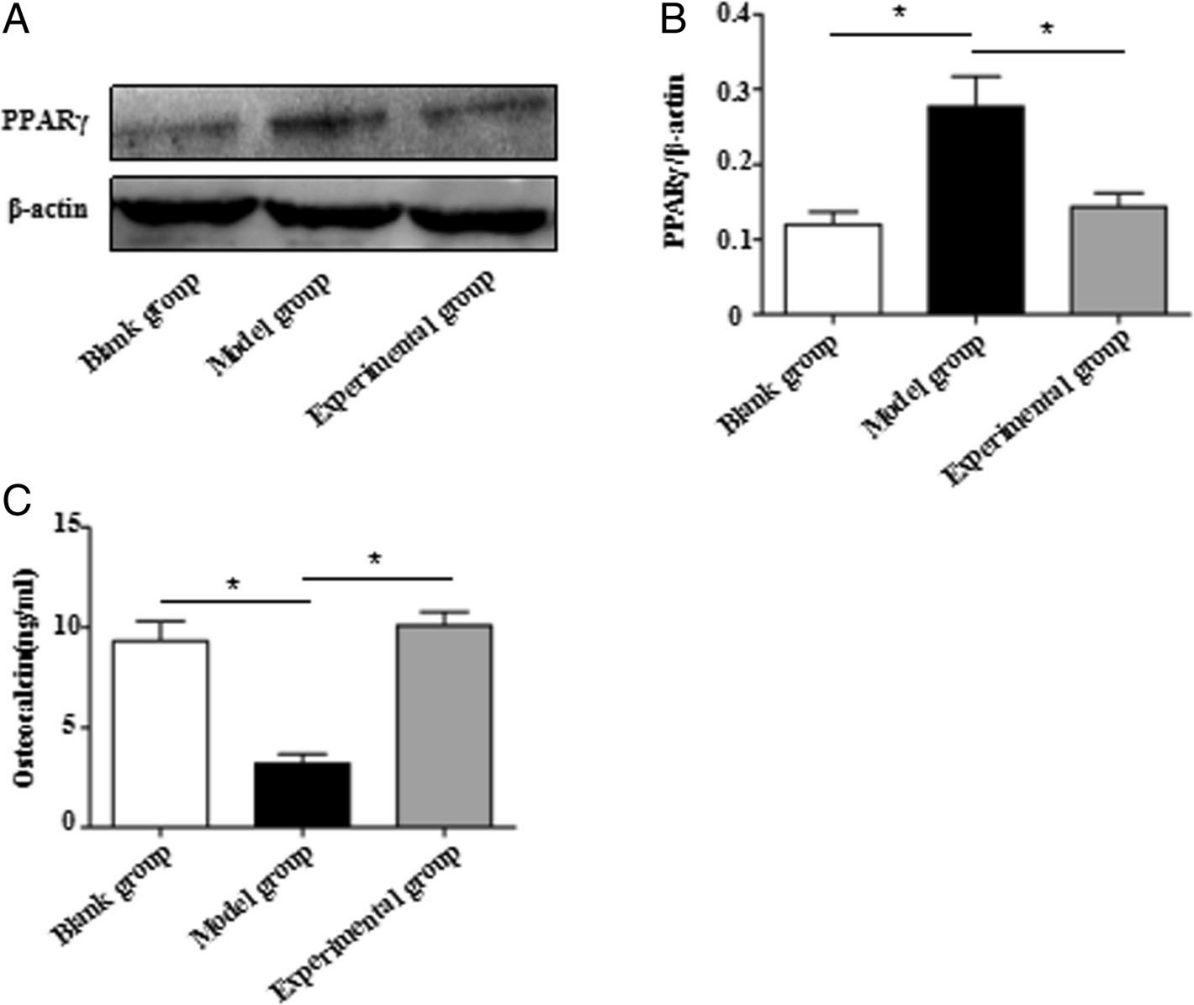
Results of western blot and ELISA. **a**, **b** The result of western blot showed that PPARγ protein was significantly decreased by psoralen. **c** The ELISA result also showed that psoralen increased osteocalcin protein in experimental group

The result of western blot showed that PPARγ protein was significantly decreased by psoralen (Fig. 4a, b). The ELISA result also showed that psoralen increased osteocalcin protein in experimental group (Fig. 4c).

## Discussion

At present, the etiology of ANFH is relatively clear, which is due to prolonged and/or high-dose use of glucocorticoid-induced osteonecrosis of the femoral head. SANFH is a metabolic disease that occurs due to the use of glucocorticoid drugs, leading to impaired blood supply to the femoral head and death of bone cells and bone marrow composition, which in turn lead to structural change, collapse of the femoral head, and articular dysfunction [8]. But in its pathogenesis, the medical profession has not yet had clear conclusions and the following several theories have been widely recognized.

### Theory of lipid metabolic disorders

Prolonged high-dose use of hormones can increase blood lipids and cause hyperlipidemia, leading blood tends to be stagnated. The fat embolism situation of the whole body is serious, and the femoral head tiny arteries form adipose suppository, which directly causes the partial blood stasis [9]. The bone marrow cells are occupied by adipose cells, and the adipose cells fuse into pieces, inducing the death of marrow-derived cells, resulting in ANFH.

### Theory of intraosseous pressure increasing

As early as 1981, Bünger et al. suggested that the increase of intraosseous pressure is the pathogenesis of ANFH [10]. The medical profession believed that the increase in intraosseous pressure was due to excessive accumulation of adipose cells in the bone marrow, so that the pressure in the blood vessels increased, blood vessels spasm caused ischemia, and then form ANFH. But at the same time, some scholars pointed out that the increase in intraosseous pressure in the middle and late stage of the disease was a secondary change, and early ANFH did not appear in the intraosseous pressure phenomenon. Although there is no obvious evidence that indicates that the increase in intraosseous pressure is the primary or secondary change of ANFH, the theory of intraosseous hypertension has been universally recognized.

### Theory of osteocyte apoptosis

The medical profession generally believes that the use of glucocorticoid in excess of conventional doses can cause ANFH. Studies have shown that high-dose applications of glucocorticoids can decrease the activity of osteoblasts and increase the apoptosis of mature bone cells. The number of osteoblasts decreased significantly, and the trabecular bone was sparse, which resulted in osteoporosis [11]. Eerhardt et al. suggested that the staining section of rabbits with SANFH model could be observed under the light microscope, and the trabecular bone of the subchondral bone in the femoral head became thinner. Apoptosis of bone cells was prevalent in special staining around the trabecular bone [12].

### Osteoporosis theory

The negative effects of long-term use of glucocorticoids include osteoporosis. Glucocorticoids can overdecompose proteins and reduce their synthesis, resulting in the thinning of the trabeculae bone, at the same time against vitamin D, and reducing calcium absorption in the gastrointestinal tract, thus causing large amounts of calcium to be excreted. Taking large doses of steroid-like drugs are prone to become bone hyperplasia, which can reduce the number of trabecular bone and osteoblasts, thus lead to osteoporosis, and ultimately lead to the collapse of the femoral head bearing area, and even necrosis [13]. In addition, in the process of differentiation of bone marrow stromal cells into adipocytes, hormones play a certain role in inducing the reduction of differentiation of osteoblasts. These are the traditional Chinese medicine factors that lead to osteoporosis and ANFH.

At present, the treatment methods of SANFH are diversified and the indications are various. And the treatment of this disease is mainly divided into non-surgical therapy and surgical therapy, and surgical therapy can be broadly divided into two categories: minimally invasive and joint replacement. The medical profession adopts treatment options mainly based on different indications and patient wishes and their physical and economic conditions. But it is aimed to improve the quality of life of patients. The hip joint is the main weight-bearing joint in the human body, and to reduce or avoid the hip joint weight is the main concern in the course of treatment, so as to obtain the maximum clinical effect. The surgical treatment has made a great progress with the increasing incidence of SANFH and development of modern medical research. Depending on the progression of the course, different methods of operation can be chosen. At present, the commonly used surgical methods in clinical practice are as follows: (1) the ARCO classification determines that the femoral head has not yet collapsed and can be conducted femoral head drilling decompression plus bone graft or femoral head drilling decompression plus tantalum rod support surgery. The purpose is to delay the collapse of the weight-bearing area of the femoral head, even without collapse. The operation is simple and widely used in young and middle-aged people. (2) Bone flap transplantation surgery is used relatively less in recent years; it is mainly used to promote bone flap and blood vessel crawling and the growth of new bone and to delay the time of joint replacement. (3) One of the new surgical methods in recent years, stem cell transplantation surgery, is also popular, but because of its high technical requirements and high cost, results in the range of use is very small, but the scope of surgery and the operation can promote the repair of ANFH, so the experimental research is also increasing. (4) Joint replacement is currently the most widely used surgical method in middle-aged and old people; the surgical technique is mature, the cost is relatively low, and the quality of life improved obviously. For the use of traditional Chinese medicine, every theory has its own unique insights. But generally speaking, tonifying liver and kidney and strengthening bones and muscles have been generally recognized [14]. In addition, long-term application of hormones leads to hyperlipidemia, a systemic fat embolism. And small artery lumen at the end of the femoral head cartilage is rare [15]. The fat clumps that adhere to the inner wall of the vessel form fat embolism. Bone marrow cells are occupied by fat cells. Fat cells fuse into pieces, resulting in hemopoietic cells in the bone marrow dead. Based on this theory, many factors, such as lipid-lowering drugs, anticoagulant blood, and vascular relaxant, which are used to control the necrosis of the femoral head caused by the proliferation of adipocytes, are widely used in clinical treatment [16]. However, there is no clear and unified view on the usage, dose, and the application time.

PPARγ mRNA mainly plays a role in regulating fat, so it has great significance in the process of adipogenesis of cells in blood vessel and bone marrow [17, 18]. Excessive differentiation of the adipose tissue leads to sparse trabecular bone, where bone fat cells are multiplied, resulting in osteoporosis, which can lead to minor fractures of bone in severe cases [19, 20]. Through the experimental study on the adipogenic effect of bone marrow stromal stem cells from osteoporosis rats, Liu et al. [21] found that regulating and inhibiting the expression of PPARγ mRNA can effectively reduce the incidence of osteoporosis. At the same time, it also confirmed that the low expression of PPARγ mRNA could reduce the risk of small fracture and reduce ANFH.

Osteocalcin mRNA is one of the most sensitive indexes in the process of osteogenic differentiation [22]. The increase of its activity can accelerate the process of ALP activity combined with it and thus promote the bone formation of osteoblasts [23]. On the other side, the combination of osteocalcin and calcium ions has a positive effect in promoting bone deposition and bone growth. Peng et al.’s exploration from the side reflects the activity of osteocalcin having a positive correlation with osteoblast differentiation and osteoclast apoptosis [24]. Zhu et al. believe that one of the bone secretion hormones is osteocalcin, which regulates the pathogenesis of osteoblasts and osteoclasts and regulates adipocytes by converting osteoclasts into blood [25]. Therefore, the high expression of osteocalcin can effectively reduce the differentiation of adipocytes, increase bone matrix, and strengthen sclerotin [26, 27].

## Conclusions

In clinic, because of the treatment of diseases, many patients have to take a lot of hormones for a long time, which leads to SANFH. After discovering the effect of *Psoralea* on the treatment of ANFH through animal experiments, we applied the dialectical thinking of traditional Chinese medicine and took this drug as the main drug (sovereign drug) combined with other traditional Chinese medicine to constitute the traditional Chinese medicine prescription for the prevention and treatment of SANFH. The medicine in the prescription was decocted and boiled together and taken by the patient for different times depending on the patient’s condition. The results were satisfactory, especially for patients with early SANFH.

In this experiment, compared with blank group, the relative expression of PPARγ in the model group was significantly increased, and the expression of osteocalcin was significantly reduced; compared with model group, the PPARγ of the experimental group was significantly decreased and the expression of osteocalcin was significantly increased. This experiment confirms that psoralen can reduce the adipogenesis of cells in bone marrow, promote the deposition of calcium, prevent osteoporosis, and play a positive role in the rehabilitation of ANFH.

## Abbreviations

ANFH: Avascular necrosis of the femoral head
SANFH: Steroid-induced avascular necrosis of femoral head
TBA: Trabecula bone area
